# Perinatal Gene Transfer to the Liver

**DOI:** 10.2174/138161211797247541

**Published:** 2011-08

**Authors:** Tristan R McKay, Ahad A Rahim, Suzanne M.K Buckley, Natalie J Ward, Jerry K.Y Chan, Steven J Howe, Simon N Waddington

**Affiliations:** 1 William Harvey Research Institute, Queen Mary University of London, London, UK; 2 Institute for Women’s Health, University College London, London, UK; 3 Experimental Fetal Medicine Group, National University of Singapore, Singapore; 4 Institute of Child Health, University College London, London, UK

**Keywords:** Fetal gene therapy, *in utero* gene therapy, neonatal gene therapy, lysosomal storage disease, mucopolysaccharidosis, hemophilia, glycogen storage disease, liver, viral vector.

## Abstract

The liver acts as a host to many functions hence raising the possibility that any one may be compromised by a single gene defect. Inherited or de novo mutations in these genes may result in relatively mild diseases or be so devastating that death within the first weeks or months of life is inevitable. Some diseases can be managed using conventional medicines whereas others are, as yet, untreatable. In this review we consider the application of early intervention gene therapy in neonatal and fetal preclinical studies. We appraise the tools of this technology, including lentivirus, adenovirus and adeno-associated virus (AAV)-based vectors. We highlight the application of these for a range of diseases including hemophilia, urea cycle disorders such as ornithine transcarbamylase deficiency, organic acidemias, lysosomal storage diseases including mucopolysaccharidoses, glycogen storage diseases and bile metabolism. We conclude by assessing the advantages and disadvantages associated with fetal and neonatal liver gene transfer.

## 1 INTRODUCTION

The importance of the liver has been recognised for thousands of years. During the reign of the Egyptian empire the god Imsety was believed to protect the canopic jar that held the liver of the deceased. At this time, the Egyptians thought that the liver was the seat of emotion. Modern medicine has recognised the liver as being the seat of many fundamental functions including; amino acid metabolism, transamination, plasma protein synthesis, storage of glycogen, and bile production. During the 1990s gene therapy went from being vaunted as a panacea to being vilified as snake oil. However the first decade of this new millennium has witnessed a ‘renaissance’ within the field as, case by case, clinical successes of gene therapy have been demonstrated. 

In this review we first consider the main vector classes that have been used for liver gene transfer in fetal neonatal models, namely adenoviral vectors, retroviral vectors (specifically gamma retroviral and lentiviral vectors), adeno-associated viral vectors and non-viral vectors. We proceed to detail classes of disease which have been studied in preclinical models including coagulopathies, urea cycle disorders, organic acidemias, lysosomal storage diseases, glycogen storage diseases and disorders of bile metabolism. Finally we overview the advantages, disadvantages and questions facing the concept of fetal and neonatal gene therapy including risks of genotoxicity, the diseases which may benefit from early intervention, and the choice of vector depending upon the disease. For a broader, rather than a liver-based, perspective on the concepts and technologies underlying fetal gene transfer the reader is directed to recent and extensive reviews on the subject matter [1-5].

## 2 VECTOR SYSTEMS FOR FETAL AND NEONATAL GENE TRANSFER

### 2.1 Adenoviral Vectors

Adenovirus-based vectors have been used for gene delivery to a wide range of cells and organs and have been implemented in many preclinical studies. Adenoviral vectors contain a DNA payload that, when delivered, remains episomal within the nuclei of infectedcells. Fifty-one known human adenovirus serotypes are categorised into six subgroups, A-F. Most commonly, serotype 5 (Ad5) has been used for preclinical and clinical trials. However, enthusiasm for its use has been blunted by the high prevalence of pre-existing anti-Ad5 immunity in mice, rhesus monkeys and humans [6]. Elucidating the complex interaction between receptors, capsid elements, blood cells and proteins is critical to its adoption in clinical settings. In 1993, Ad5 was shown to depend upon α_v_β_3_ and α_v_β_5_ integrin for virus internalisation [7]. In 1997, the primary receptor for the Ad5 fiber was shown to be the coxsackie and adenovirus receptor (CAR) [8, 9]. However these receptors did not account for the high efficiency for Ad5 in binding to the liver. Recently we [10], and others [11, 12], have shown that coagulation factor X (FX) acts as a molecular bridge between the adenovirus hexon hypervariable regions and cell surface ligands. This interaction is the major mediator for *in vivo* infection of hepatocytes in rats and mice. By administering warfarin [13] to deplete all vitamin K-dependent coagulation factors or by injection of specific pharmacological inhibitors of FX, such as factor X-binding protein, adenovirus infection can be profoundly inhibited in the liver and substantially reduced in other organs including the lung, heart and spleen [10, 14]. Liver infection can also be inhibited by genetic modification of Ad5 hypervariable regions 5 and 7 [15]. 

For anti-cancer studies, adenoviral vectors that are capable of replication in selective environments have been developed [16, 17]. However, for a high proportion of preclinical and clinical studies, first and second generation adenoviral vectors have been employed where viral genes including E1 and E3 have been deleted. These vectors are incapable of independent replication but still express other viral genes at a low level that contributes to toxicity and transient expression, which are often seen following application of these vectors. To reduce this toxicity and increase the vector payload further, fully-deleted “gutless” vectors have been developed. These are known as helper-dependent adenoviral vectors, contain no viral genes and therefore encode no viral proteins. These vector iterations are reviewed by Alba and colleagues [18].

### 2.2 Retroviral Vectors

Retrovirus virions contain two RNA strands of genome that are associated with a reverse transcriptase molecule within a protein capsid and surrounded by a membrane coat. On infection, the virus initiates reverse transcription of the RNA genome to DNA that integrates into the host cell genome facilitated by the virally transcribed integrase. The lipid bilayer coat, budded from the host cell membrane, displays virally transcribed receptors that determine tropism. Gene delivery vectors have been developed based upon several genera of the *Retroviridae* family including Avian leukosis sarcoma virus (genus Alpharetrovirus) [19], human foamy virus (genus Spumavirus) [20] but most commonly Moloney murine leukemia virus (MLV) (genus Gammaretrovirus) and Human Immunodeficiency Virus type (HIV)-1 (genus Lentivirus) [21]. The application of retroviridae as gene transfer vectors was limited in the early stages by their physical instability, resulting in reductions of active half-life and an inability to substantially increase viral titre by standard methodologies, such as caesium chloride centrifugation or ultracentrifugation. Furthermore, viral tropism was often limited to the tissue specificity of the host virus. Pseudotyping virions with the envelope glycoproteins of an alternative virus has served to somewhat resolve both of these issues. Witte *et al*. first showed that MLV gammaretroviruses could be successfully pseudotyped with the vesicular stomatitis virus glycoprotein (VSV-G) in 1977. This increased virion stability, improving titres, as well as substantially broadening tropism. In the mid-1990s, Miyanohara *et al*. demonstrated that injection of VSV-G-pseudotyped MLV-based vector into the neonatal mouse liver resulted in strong marker gene expression with little expression in other organs [22]. Pseudotyping has become widely utilised as a process of increasing viral titre as well as broadening tropism (VSV-G, Ebola, Mokola and lymphocytic choriomeningitis virus G protein (LCMV) [23] or targeting specific tissue-types *in vivo* (influenza HA to the lung, rabies virus glycoprotein to neurons) although many still retain a strong liver tropism. Interestingly, pseudotyping with the gp64 envelope protein of the insect baculovirus, *Autographa californica, *confers increased hepatocyte tropism [24] highlighting both the flexibility but equally the complexities underlying viral pseudotyping.

Such principle advances have enabled the efficacy of gammaretroviruses and lentiviruses as gene transfer vectors in both animal and human studies, as have further vectorological manipulations outlined with reference to specific vectors below. Concurrently, this has necessitated parallel progress in terms of safety, especially when using vectors based on human pathogens such as HIV-1.

#### 2.2.1 Gammaretroviruses

Gammaretroviruses, near universally represented by MLV based vectors, were first successfully used to transduce mouse hematopoietic progenitor cells capable of partially reconstituting bone marrow *in vivo* after positive selection of a marker transgene by Eglitis *et al*. in 1985 [25]. These MLV vectors were gutted of all coding sequences and contained only viral Long Terminal Repeats (LTRs) and an RNA packaging signal. MLV gag/pol and the endogenous MLV envelope were expressed *in trans* in producer cells. Since this seminal study, safety has been improved somewhat by introducing self-inactivating (SIN) LTRs and tropism increased by pseudotyping, largely with VSV-G [26]. Nevertheless the use of vectors pseudotyped with ecotropic, amphotropic and gibbon ape leukemia virus (GALV) is common. In practice today’s gamma retroviral vectors differ very little from those described in 1985.

#### 2.2.2 Lentiviral Vectors

HIV-1 based vectors have been by far the most commonly used lentiviral vector for gene transfer since the mid 1990s. They are reported to be able to transduce both dividing and non-dividing cells due to the nature of the viral integrase [27] and the presence of the central polypurine tract (cPPT) but early HIV vector formats transduced quiescent hepatocytes relatively poorly. However, incorporation of additional elements such as the woodchuck hepatitis virus post-transcriptional regulatory element (WPRE) enhanced vector production [28]. Lentiviral vectors have also been derived from equine infectious anemia virus (EIAV) [29], feline immunodeficiency virus (FIV) and simian immunodeficiency virus (SIV). A high tropism for liver has been demonstrated following intravenous injection into fetal mice with EIAV [30] and neonatal mice with FIV [31]. 

#### 2.2.3 Retroviral Integration

Although the efficient and persistent expression achieved by retroviral vectors can be partly ascribed to their ability to integrate into the host genome, the downside is that insertion of a transgene in the host genome in the vicinity of a tumor suppressor gene or proto-oncogene may result in a malignant transformation of the cell. Moreover, it has been shown that retroviruses do not integrate in a completely random manner. For example gammaretroviruses preferentially integrate into the 5’ flanking region of expressed genes near transcription start sites [32], whereas HIV vectors integrate across the whole transcriptional unit. In response to the perceived dangers of these vector systems, a generation of non-integrating retroviral vectors have been developed. These vectors take advantage of the fact that following reverse transcription, instead of integrating, the DNA can assume a circular episomal format containing either one or two LTRs [33]. This form is very stable and provides long term expression in postmitotic tissues. To prevent integration from occurring vectors have been synthesised contain a variety of mutations including those at critical residues of the viral integrase and those in the nucleotides which mediate binding of the integrase to the vector genome [34]. Very recently, integrase-deficient lentivirus vectors (IDLV), injected into adult mice, have been shown to result in long-term expression of human coagulation factor IX and to induce strong and antigen-specific immune tolerance, despite being up to 15-fold less efficient than the integrating format of the vector [35].

### 2.3 Adeno-associated Viral (AAV) Vectors

Adeno-associated virus is a member of the parvovirus family and is one of the smallest DNA viruses. Wild type adeno-associated virus serotype 2 contains a single-stranded linear stretch of DNA comprising two genes which encode four replication proteins and three capsid proteins. In the absence of helper adenovirus or herpes simplex virus, wild type AAV establishes latency by integrating into human chromosome 19. Vector derived from AAV virus is a “gutless” design in that all viral genes are replaced with vector payload, flanked by inverted terminal repeats (ITR) which are the only elements necessary for packaging the gene of choice into the vector. AAV vectors have been reviewed by Greiger and Samulski [36]. AAV serotype 2 was the archetypal vector however, since then, numerous further serotypes have been identified and many more unique isolates have been derived from humans and nonhuman primates [37, 38]. The vector genome is a single-stranded DNA, therefore second strand synthesis and annealing are required to generate a double-stranded DNA which can serve as a transcription template [39]. Since this was thought to be a major rate-limiting step in AAV-mediated gene expression, efforts were made to generate self-complementary vectors. These vectors contain self-complementary molecules of DNA that can refold into double-stranded DNA templates for expression once within the cell. They have shown to be between 5 and 140-fold more efficient than single-stranded counterparts [40, 41]. However one disadvantage is that the AAV payload is approximately 4.7kb therefore a self-complementary AAV vector is limited to carrying a gene plus promoter of only around 2.3 kb. A second concern, in terms of liver gene transfer, is that a recent report describes that self-comple-mentary vectors induce a substantially stronger TLR9-mediated innate immune response compared to single-stranded format vector [42].

Lipshutz and colleagues were early pioneers in using AAV to study gene transfer to the fetal or neonatal liver. In 2001, they used AAV2 to deliver the light-emitting protein luciferase to the peritoneal cavity of day 15 gestation mice. This resulted in strongest transduction of liver compared to other visceral organs such as spleen, kidney and intestine. Although expression, as measured by whole body bioluminescent imaging, declined approximately 1000-fold in the first month, expression was still detectable at six months of age [43]. 

The efficiency that can be attained using AAV vectors was recently put to good use by Paulk and colleagues, working towards a major goal of gene therapy – gene repair as opposed to gene supplementation. They used a mouse model of inherited tyrosinemia type I which carries a single point mutation rendering it deficient in fumarylacetoacetate hydrolase (FAH). This disease causes accumulation of toxic metabolites in the liver and the renal proximal tubule and causes death to the specific affected cells. The mouse model is an accurate, telescoped model of the human disease and serves as an excellent model for gene repair as corrected hepatocytes have a survival advantage compared with untreated cells. The authors injected AAV2 and AAV8 vectors carrying wild-type genomic sequence for repairing the mutant *FAH *gene into neonatal and adult mice. Gene repair was successful at both ages of administration using AAV2 and AAV8, though more effective with the latter [44].

### 2.4 Non-viral Gene Transfer

Oft-cited concerns over the use of viral vectors include insertional mutagenesis (by retroviral vectors), immunogenicity of viral components and limitations in vector payload size. This has, historically, motivated development of non-viral vectors in which DNA is delivered using polymers, polycations and liposomes either singly or, sometimes, in combination. A major failing of many non-viral gene therapy protocols has been the rapid elimination of expression as a consequence of gene silencing or dilution due to a lack of integration. Nevertheless, some studies have shown that with thoughtful incorporation of promoter and regulatory elements, long-term expression of luciferase marker gene [45] and the therapeutically relevant transgenes factors VIII and IX [46] can be achieved in adult mice following rapid, high volume intravenous injection. More recently, Wong and colleagues achieved strong, albeit transient, levels of marker gene expression under the control of the human α-1-antitrypsin promoter in hepatocytes of neonatal immune competent mice using polyethylenimine (PEI) as the delivery agent [47]. 

## 3 DISEASE TARGETS FOR FETAL AND NEONATAL GENE THERAPY

### 3.1 Coagulation Disorders - Hemophilias A and B

The liver produces many of the factors of the coagulation cascade including; fibrinogen (factor I), prothrombin (factor II), factors V, VII, VII, IX, X and IX, protein C and antithrombin [48, 49]. This cascade comprises a series of proteases and cofactors which control the generation of fibrin and thus, permits blood clotting at sites of injury but maintains an anti-coagulant state in the absence of vessel injury [50]. Mutations in the X-linked genes encoding coagulation factors VIII and IX are responsible for hemophilias A and B, respectively. Patients suffer from recurrent haemorrhage particularly into muscles and joints. The latter ultimately can lead to inflammation of the synovium and degenerative arthritis of one or more joints; the disease affects nearly half a million people in the world. In developed countries current treatment involves injection of the missing coagulation factor. These were initially obtained by plasma cryoprecipitiation but blood contaminated with HIV and hepatitis virus resulted in infections that devastated the hemophilia community. Over the past two decades recombinant coagulation factor protein has been used. Although this treatment has resulted in a great improvement in quality and duration of life, recombinant factor is incredibly expensive, has a relatively short half-life requiring administration two or three times weekly and is not readily available in developing countries. Continual protein replacement does not completely abolish the prospect of developing hemophilic arthritis. Moreover, a significant proportion of patients develop inhibitory antibodies against the injected protein which puts them at grave risk of fatal hemorrhage [51]. Many preclinical studies have been performed in small and larger animal models for treatment of hemophilia. Moreover, several of these studies have resulted in clinical trials [52]. AAV vectors, which have a strong tropism for liver, have shown the greatest promise for clinical application. AAV2 delivery to humans with severe hemophilia B resulted in expression of therapeutic factor IX concentrations for up to eight weeks [53] before an anti-capsid immune response eliminated those hepatocytes that had been infected with the AAV vector [54]. In a separate series of studies, self-complementary AAV8 containing human factor IX under the control of a synthetic liver-specific promoter was shown to establish prolonged high levels of gene expression when administered to mice and non-human primates [55]. This vector has been applied recently in a clinical trial where expression in one patient has risen from baseline to 1.5-2% and has maintained this level for more than 5 months [56]. 

The justification for treating hemophilia in the womb or in the neonatal period may not be immediately apparent; only rarely do newborns suffer life-threatening extracranial or intracranial bleeding [57] and this is generally managed well using coagulation factor supplementation once diagnosis is confirmed. However, the mean age at which children with severe haemophilia experience their first bleed into a joint is 23 months [58], most likely because they become highly active and mobile and, therefore, begin to exert increased pressure on joints. Once joint damage has occurred as a result of recurrent bleeding, secondary prophylaxis can limit, but not prevent ongoing joint damage. This, therefore, vindicates a strategy for very early treatment. Retrospective analyses have revealed that regular prophylaxis with coagulation factors is able to reduce physical impairment from hemophilia arthropathy [59]. Moreover, there is evidence that early commencement of prophylaxis and the avoidance of intensive treatment periods may reduce the risk of inhibitor development in the patient [60]. 

Ponder and colleagues have performed numerous and extensive studies on the delivery of factors VIII and IX during the neonatal period using gene therapy. In an early study, neonatal mice received intravenous injection of an MLV gamma retroviral vector expressing canine factor IX. This resulted in >100% normal levels of expression (5 µg/ml) for up to a year. The same vector, injected into three newborn hemophilia B dogs resulted in cFIX expression of between 12-36% [61]. In an advancement of this study, the same group demonstrated that neonatal FIX delivery by retrovirus induced immune tolerance to the transgenic protein and that this was dependent upon having gene expression above a threshold. Tolerance was demonstrated by challenging the neonatally-injected mice with FIX protein [62]. The group then turned their attention to delivery of canine B domain-deleted factor VIII to neonatal mice and dogs. Hemophilia A mice and dogs expressed cFVIII concentrations of 139% and 116% of normal values for more than 18 months; no anti-cFVIII antibodies were detected [63]. However, mirroring their FIX study, they observed that higher titres of FVIII retrovirus vector resulted in immune tolerization whereas mice which received low doses of vector developed antibodies before or after challenge with FVIII protein [64]. In an earlier study, Vandendriessche and colleagues had employed an MLV gamma retroviral vector to deliver human factor VIII to neonatal hemophilia A mice. They observed prolonged FVIII production in 6 out of 13 mice injected for up to at least 14 months and four of the mice expressed physiological or supraphysiological concentrations of FVIII. Interestingly, those that did not express FVIII developed anti-FVIII antibodies [65]. 

**Fig. (1):**
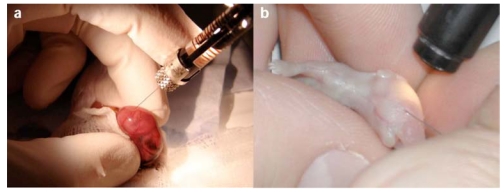
**a**. Injection into the mouse fetus following laparotomy of the mother **b**. Injection into the circulation of the neonatal mouse via the superficial temporal vein.

Fetal or neonatal gene delivery of factors VIII and IX has not been limited to gamma retroviral vectors. We demonstrated that HIV-1 based lentiviral vectors could be used to deliver human factor IX to hemophilia B mice following fetal injection (Fig. **1a**) to the circulation via the vitelline vessel. This resulted in 9-16% levels of normal human factor IX levels in the circulation for up to at least 14 months. Coagulation assays demonstrated near-normal levels of clotting function [66]. Very recently we used a lentiviral vector expressing a codon-optimised human factor VIII cDNA sequence to cure neonatal hemophilia A mice following intravenous injection by the superior temporal vein (Fig. **1b**). Eighteen mice which received different variants of FVIII expression cassette expressed >200% normal levels of human factor VIII for up to at least 250 days [67].

The use of gamma retroviral or lentiviral vectors would seem highly appropriate for gene delivery to the fetal or neonatal liver since the vector genomes integrate and, therefore, multiply in parity with the large increase in cell number in the growing liver. However, concerns over retrovirus-associated haematopoietic malignancies arising in the X-SCID clinical trial [68] and EIAV lentivirus-associated hepatocellular carcinoma following administration to fetal and neonatal mice [23] vindicates the application of episomally maintained vector formats. One of the rationales underlying the use of these vectors is that very early gene expression may facilitate induction of immune tolerization to the transgenic protein and that a booster injection of gene therapy vector could be performed once the liver was approaching or had achieved its adult mass. 

In 1999 Lipshutz and colleagues used a second-generation adenoviral vector to deliver mouse factor VIII to the fetal hemophilia A mouse by intraperitoneal injection at D15 gestation. They observed expression of 50% normal levels of mouse FVIII in the circulation 2 days after birth but this declined rapidly to undetectable levels by 21 days [69]. We administered a helper-dependent adenovirus vector to deliver hFVIII to the fetal (non-hemophilic) mouse at 15 days gestation. We measured relatively high expression, of 20% normal human levels, ten days after injection however this declined to nearly undetectable levels 37 days after injection [70]. Lipshutz and colleagues revisited this concept recently, administering helper-dependent adenovirus to neonatal hemophilia A mice to deliver human factor VIII. Three days after injection hFVIII concentrations reached 649% of normal human levels yet, again, declined rapidly in the first 28 days of life. However, thereafter, levels stabilised to around 4% for the following 16 months. This expression was sufficient to induce and maintain immune tolerization to human factor VIII and permitted a booster injection to these mice in adulthood, resulting in high (≈40%) and sustained (>3 months) expression of the clotting protein [71].

In 2002 we used an AAV2 vector to deliver human factor IX to the liver of fetal mice by injection via the vitelline vessel. At that time the available titres of AAV were such that very low concentrations of human factor IX (<1%) could be detected in mouse plasma ten days after injection [72]. Fortunately, in the subsequent years AAV-mediated gene delivery to the fetal or neonatal liver has been applied to much better effect. Sabatino and colleagues performed an elegant study where they injected AAV1 and AAV2, carrying human factor IX, to fetal, neonatal and adult mice. Fetal or neonatal administration of AAV1 was able to induce immune tolerance to human coagulation factor [73]. Very recently we have administered self-complementary AAV8 carrying human FIX (the same as is currently being used in the clinical trials mentioned above [56]) to mice at D15 gestation. Consistent with the other studies, we observed a dramatic loss in gene expression from >65% of normal hFIX levels on the day of birth to undetectable levels at around 80 days. Interestingly, hFIX expression began to re-appear at 200 days and up to at least 450 days [56]. 

This same vector has also been studied in larger animals. Sheep provide a useful model of human pregnancy; they have a consistent gestation of 145 days and the development of the fetus, particularly the immune system, is similar to that in humans. Using ultrasound guidance it has been feasible to administer gene transfer vectors to the circulation of the fetal sheep at different gestational ages [1]. Recently, David and colleagues administered AAV8-hFIX to early (around 60 days) and late (around 100 days) gestation fetuses. Following late gestation injections, blood concentrations of hFIX were particularly high, exceeding 30% of the concentration found in normal humans. In two cases, even at six months hFIX expression was maintained at around 1%. No antibodies to either capsid or FIX were detected following vector administrations. However, an immune response to hFIX was induced by immunising the lambs with FIX protein in combination with an immune (Freunds) adjuvant [74]. In a parallel study, Mattar *et al*. injected the same vector form, or one pseudotyped with serotype 5 capsid, to the late gestation fetal macaque and observed widespread liver transduction with both vectors. Although there was an initial dramatic fall in FIX expression, it was stably maintained at levels between 7 and 111% of normal levels for between 2 and 22 months. Anti-capsid antibodies could be detected after AAV5 delivery and less so following AAV8 however these antibodies did not eliminate expression [75].

### 3.2 Coagulation Disorders - Early and Lethal

Unlike hemophilias A and B, mutations resulting in complete deficiency of some coagulation factors result in very severe, very early onset disease phenotypes. 

Coagulation factor X plays a fundamental role in the coagulation cascade. It associates with factor V on the cell membrane to form part of the prothrombinase complex that converts prothrombin to thrombin. Inherited factor X deficiency is rare and in clinical presentation is one of the most serious of the bleeding disorders. The more severe cases present at birth with bleeding from the umbilical stump or, more gravely, in the central nervous system [76]. Apart from lethality, in some cases these bleeds result in severe neurological sequelae such as paralysis, blindness and seizures [77]. In the late 1990s, a gamma retroviral vector was designed to carry human factor X under the control of the liver specific α-1-antitrypsin promoter. This was injected into adult rats and resulted in levels of expression of 10-43% in four rats. However, a partial hepatectomy was required to achieve efficient liver transduction and several rats generated anti-FX antibodies [78]. To overcome these hurdles the group administered AAV2-FX to neonatal mice. Although lower levels, of approximately 7%, were detected they were achieved without partial hepatectomy and persisted for more than 14 months with no evidence of immune response [79]. 

Coagulation factor VII interacts with tissue factor at the site of vascular injury to form a serine protease, FVIIa, which is pivotal for activation of coagulation. Therefore, congenital FVII deficiency, the most common autosomal recessive bleeding disorder, is characterised by a bleeding diathesis that is extremely severe or lethal in at least 20% of patients with a homozygous or compound heterozygous genotype. These individuals generally have plasma FVII activity below 1% of normal and commonly (approximately 60%) present with life threatening haemorrhage within critical organs such as the central nervous system during the neonatal period although their *in utero* development is normal [80, 81]. Promising preliminary data has been generated from experiments using self-complementary AAV5 vectors expressing human factor VII. Ultrasound-guided injection into the peritoneal cavity of three fetal macaques at 0.9 years gestation conferred therapeutic expression of human FVII at the time of birth (20% of normal levels) with expression being maintained above 5% of normal levels for at least 2 months postnatal [82].

Deficiency of *A* *D*isintegrin *A*nd *M*etalloprotease with *T*hrombospondin (ADAMTS13) results in thrombotic thrombocytopenic purpura (TTP). More than 50% of humans with severe ADAMTS13 deficiency present at birth and the disease is characterised with recurring hemolytic and thrombocytopenic crises. Cerebral vascular incidents frequently result in neurological damage and there is a high risk of kidney failure following massive hematuria during hemolytic crises. The first descriptions of inherited TTP, in 1953, included three affected siblings, two of which were born jaundiced and subsequently died of bowel bleeding and haemorrhage at 4 days and 2 years of life. Plasma therapy, every 2-3 weeks, has formed the mainstay of treatment for many years. However, patients with long intervals between crises are not placed on regular plasma therapy yet occasionally suffer from central nervous system and renal involvement. Recombinant ADAMTS13 exists but is not used for clinical treatment [83], therefore early gene therapy may be a useful therapeutic approach. Niiya *et al*. used a lentiviral vector to deliver ADAMTS13 (and a truncated variant) to mice deficient in that gene. These mice have a prothrombotic phenotype characterised by the accumulation of excessively large von Willebrand Factor (vWF) multimers in the plasma and enhanced platelet-endothelial cell adhesion. The authors injected vector at 8 and 14 days gestation into the amniotic fluid and vitelline vessel respectively, the latter being associated with a lower rate of fetal mortality and targeted transduction of liver tissue. Treated mice exhibited a reduction in the size of vWF multimers and a significant prolongation of ferric chloride-induced carotid arterial occlusion time [84]. 

### 3.3 Urea Cycle Disorders

The urea cycle, a process of breakdown of amino acids and elimination of ammonia in the form of urea, takes place primarily in the liver and to a lesser degree in the kidney. Genetic disorders of the urea cycle typically manifest as high ammonia levels and disturbed amino acid metabolism. There are six well-known defects of the urea cycle that are deficiencies of the enzymes arginase, ornithine transcarbamylase (OTC), arginosuccinate synthase, arginosuccinate lyase, carbamoyl phosphate synthase and *N*-acetyl-glutamate synthase. Newborns with urea cycle disorders tend to have a normal birth weight but present within hours to days after birth with serious illness commencing with vomiting, lethargy and rapid breathing which then progresses to hyperammonemia-induced coma. Of those who survive this period through aggressive therapy, half die before school age and nearly all have severe developmental disabilities. These diseases are treated by severe protein restriction and administration of sodium benzoate and sodium phenylbutyrate which stimulates alternative nitrogen clearance mechanisms. However, patients are still prone to suffer intermittent bouts of hyperammonemia. Liver transplantation is a valid alternative to long term pharmaceutical intervention and dietary restriction that results in complete correction of the metabolic defect. However, it only halts but does not reverse the neurological damage. It has been shown that liver transplantation early in life is associated with improvement of neurological outcomes in children [85, 86]. Unfortunately, due to technical limitations and shortage of size-matched donor livers, this approach is not widely available to neonatal or infant patients and the procedure bears considerable risk [87]. In addition to these factors, a further justification for development of early intervention gene therapy is the evidence that urea cycle defects may cause neurological damage *in utero*. In one case, a neonate born with OTC deficiency died at 17 days of age and post-mortem analysis of the brain revealed lesions that, by their appearance, were more than eight weeks old. In the absence of no prenatal event to account for these lesions it was likely they arose from the genetic defect. Although it might be expected that fetal metabolic insufficiencies should be compensated by maternal metabolism, in this case the mother was a carrier, therefore she may have had partial OTC deficiency and was unable to compensate sufficiently for the fetal defect [88].

In 1990, early in the history of preclinical gene transfer technology, it was reported that an adenoviral vector encoding rat OTC was injected into a mouse model of OTC deficiency. The vector, injected intravenously into 1 day old mice, partially corrected the metabolic defect; an increase in hepatic OTC activity was detected and urinary orotic acid was reduced. OTC transcripts were detectable a year after injection and one mouse expressed >50% normal levels at 15 months. OTC-deficient mice display a phenotype of sparse fur until weaning but adenovirus-OTC reversed this phenotype in some individuals. However, results were quite variable [89]. Six years later, Morsy and co-workers used an adenoviral vector to deliver human OTC to OTC-deficient mice by transcutaneous injection into the neonatal liver parenchyma. Urinary orotic acid was reduced and there was a moderate increase in enzyme activity. However, they also reported a dominant negative effect of the endogenous mutant protein on the activity of delivered recombinant wild-type human protein. They suggested this was due to the formation of inactive enzyme heterotrimers containing mutant and wild type subunits [90]. 

Gene therapy in adult OTC-deficient mice has been significantly more successful with long-term correction of the defect being reported in several studies using helper-dependent adenovirus [91, 92] and AAV [93] vectors. In a comparative study, Cunningham and colleagues injected AAV vector into adult and neonatal OTC-deficient mice. Following adult delivery phenotypic correction was prolonged and complete. However, following neonatal delivery full metabolic correction was transient although modest levels of enzyme activity persisted into adulthood. The authors admitted that the severe form of the disease presents neonatally and that neonatal gene transfer would be greatly desirable [94].

Although the majority of preclinical studies have been performed using OTC-deficient mouse models, Gau and colleagues recently demonstrated proof-of-principle for correction of arginase deficiency. A helper-dependent adenoviral vector was used to deliver mouse arginase I to an arginase-deficient mouse model. A significant improvement in survival was noted, from 14 days in the untreated group to 27 days in those receiving vector. However, it was believed that the mice ultimately succumbed to hyperammonemic crisis due to arginase levels falling to less than 10% as transduced cells were diluted out by the growing liver [95].

An adenoviral vector was used to deliver human arginosuccinate synthetase to the mouse model deficient in this enzyme. Gene delivery extended the average lifespan of these affected mice from 30 hours to 16 days. Liver enzyme activity was detected at 47% twenty-four hours after vector infusion, peaking at >80% of normal levels seven days later before decreasing to 20% within 3 weeks. Despite liver enzyme levels being nearly corrected the treated mice had retarded growth, suggesting that reconstitution of liver enzyme activity was insufficient to fully reverse the disease pathology. Involvement of other organs such as kidney was, therefore, suggested [96].

It is apparent, from these studies, that correction of this class of genetic diseases requires substantial levels of activity, likely greater than 10% of normal levels. Given the failure to mediate correction of these diseases following neonatal adenovirus or AAV vector administration, it is somewhat surprising that there have been no studies using gamma retroviral or lentiviral vectors, or studies where there is sufficiently robust gene transfer using non-integrating vectors to achieve a plateau of enzyme activity at physiological levels. 

### 3.4 Organic Acidemia

Organic acidemia describes a group of diseases characterised by excretion of urinary non-amino organic acids. They usually arise from disruption of one of numerous steps in amino acid breakdown, particularly genetic deficiency in the relevant enzyme. In a somewhat similar pattern to urea cycle disorder, the infant is born well but presents with vomiting, poor feeding, lethargy, fitting and, ultimately, coma and death [97-99]. This group of diseases includes maple syrup urine disease (deficiency in the branched chain keto acid dehydrogenase) [100], propionic acidemia (deficiency in propionyl-coenzyme A carboxylase) [101], methylmalonic acidemia (methylmalonyl coenzyme A mutase) and isovaleric acidemia (deficiency of isovaleryl-coenzymeA dehydrogenase). 

For maple syrup urine disease, to minimise damage to the neonatal brain, detoxification by continuous blood exchange transfusion, hemodialysis or hemofiltration must be performed urgently. The patient is then managed by a strict dietary regime. Although most patients are expected to survive and some attend regular schools, intellectual outcome is substantially below average and metabolic crises are not uncommon. For propionic and methylmalonic acidemia the prognosis is more grave, with more severe neurological outcomes and also pathological changes in the heart and kidneys [97]. 

In 2007, Chandler and Venditti used a second-generation adenoviral vector expressing the methylmalonyl coenzyme A mutase gene under the constitutive control of a CMV promoter to treat a mouse model of methylmalonic acidemia. They administered vector into neonates by transcutaneous liver injection and direct muscle injection. They demonstrated that intrahepatic delivery improved 50% survival from less than 2 days to around 20 days. Some treated mice survived for over 7 months. Using an adenoviral vector in this model was important in order to achieve very rapid gene expression but the vector was also likely subject to loss of transient expression and/or promoter silencing which accounted for the relatively short-term correction. Moreover, the authors observed general procedure-related mortality after intrahepatic injection [102]. Three years later, the same group used an AAV vector containing a liver-specific thyroxine-binding globulin promoter in the same model system. In this study they characterised the model using many more uninjected mice (58 versus 17 previously). They demonstrated much more effective correction with 5 out of 6 mice which had received 4x10^11^ AAV genomes surviving for more than one year [103]. The improvement in survival compared with the adenoviral vector administered mice may be attributable to both the increased efficiency of transduction of the AAV vector and also the resistance to silencing of the thyroxine-binding globulin promoter compared with the CMV promoter. In 2008, Hofherr and colleagues generated both early generation and helper dependent adenoviral vectors, with or without polyethylene glycol modification, and AAV vectors, for delivery of propionyl-coenzyme A carboxylase into a mouse model of propionic acidemia. Untreated mice usually die within 36 hours of birth but the authors observed a modest, yet statistically significant increase in lifespan following intraperitoneal injection of vector [104]. In 2011 the authors joined Chandler and Venditi in using an AAV vector containing a CMV/Chicken β-actin promoter to deliver propionyl-coenzyme A carboxylase by direct intrahepatic injection. This team saw a dramatic improvement in survival, from less than 10 days (11 mice) to a 50% survival at 40 days with one mouse surviving up to at least 9 months. Significant concentrations of enzyme expression in the treated mice were also shown [101]. 

### 3.5 Lysosomal Storage Diseases

Lysosomal disorders result from defective function of a specific protein that results in lysosomal accumulation of undegraded substrate or products of catabolism that are unable to exit the lysosome. More than fifty lysosomal storage diseases are known, most of which are inherited in an autosomal recessive fashion. Lysosomes are present in all cells possessing nuclei and form part of a complex intracellular recycling system that consists of many degradation steps. Defects in lysosomal enzymes, cofactors or transport proteins may result in specific lysosomal storage disorders which can be classified and categorised by the types of storage material which accumulates. Many present in infancy or early childhood although some present with a very severe and very early phenotype, even sometimes *in utero*. Of the lysosomal storage diseases which present early, one of the first clinical presentations is non-immune hydrops fetalis – the accumulation of fluid in two or more fetal compartments. A second clinical presentation is neonatal dysmorphism. This group of diseases is reviewed extensively by Wraith [105]. Although lysosomal storage diseases have an extremely varied phenotype and may affect various organ systems including the nervous system, the haematopoietic system and muscle, numerous preclinical studies have demonstrated therapeutic efficacy from liver targeted gene therapy approaches. This success is partly based upon the application of the liver as a genetic factory for the production of the missing enzyme and has shown greatest success in those diseases where enzyme replacement therapy is most efficacious. 

#### Mucopolysaccharidoses

A subcategory of lysosomal storage diseases which has received particular attention from the gene therapy community are the mucopolysaccharidoses. This subcategory refers to a group of diseases characterised to defects in degradation of mucopolysaccharides or, more accurately, glycosaminoglycans. They include type I (Hurler syndrome), II (Hunter syndrome), III A,B,C and D (Sanfilippo syndrome), IV A and B (Morquio syndrome), VI (Maroteaux-Lamy syndrome), VII (Sly syndrome), IX (Natowicz syndrome). Consistent observations within the mucopolysaccharidoses include increased activity of β-hexosaminidase, α-glucosidase, β-galactosidase and β-glucuronidase within the liver and brain with decreased sialidase and N-acetylglucosaminyltransferase. These are reviewed by Clarke [106]. Recent technological developments have led to the production of enzyme replacement therapy for mucopolysaccharidoses types I, II and VI. Since the enzyme cannot cross the blood-brain barrier, improvement in central nervous system manifestations is not seen, although bone marrow transplantation has been shown to stabilise CNS pathology in some diseases such as Hurler Syndrome [107]. Both approaches have significant shortcomings; enzyme replacement therapy is very expensive (>300,000 USD per patient per year) and bone marrow transplantation is both expensive (>100,000 USD), requires a compatible donor and carries a 15% risk of early death. Although the liver is affected the main organs affected are the brain, heart, bone and joints. Nevertheless the liver has been targeted by gene transfer to transmogrify it into a biofactory for synthesis of the deficient enzyme. Gene therapy of these diseases has been reviewed in detail by Ponder and Haskins [108]. The majority of gene transfer studies have been performed in mouse, cat and dog models of mucopolysaccharidoses I and VII, particularly in the neonatal period. Adenoviral vector has been used in neonatal mice deficient in mucopolysaccharidosis VII [109, 110]. AAV2 has been injected into neonatal mice with mucopolysaccharidosis I [111] and neonatal MPS VII mice [112]. Alternative AAV pseudotypes have been adopted in more recent years. For example AAV8 was applied MPS II mice [113] albeit in adults, rather than neonates. An important study by Tessitore and colleagues used AAV8 for liver delivery and AAV1 for muscle delivery in neonatal MPS VII cats and rats. The authors concluded that gene expression in the liver was the most effective for disease correction [114]. Gamma retroviral vectors have been the most extensively applied for treatment of mucopolysaccharidoses, probably because the interested research groups have extensive experience with this vector, but also likely because this strategy has proved very effective following neonatal gene transfer. Gamma retroviral vector has been used in neonatal MPS I mice [115, 116], neonatal cats [117] and fetal [118] and neonatal dogs [119]. This vector class has also been applied to neonatal MPS VII mice [120] and dogs [121-124]. An *ex-vivo* gene transfer and re-implantation of fetal liver cells to mice *in utero* has also been shown to provide a modest delay in pathological changes [125].Whereas AAV vectors have resulted in a range of enzyme expression from 1% to 1700%, i.e. 17-fold over physiological concentrations, gamma retroviral vectors have achieve corrections as much as 500-fold over physiological concentrations. Achieving very high plasma concentrations appears to be important for cross-correction of systemic pathology of liver-synthesised enzyme.

In response to concerns over safety of gamma retroviral vectors, there has been a move towards use of lentiviral vectors in the field of mucopolysaccharidosis research. For example Metcalf and colleagues employed a self-inactivating lentiviral vector for delivery of α-l-iduronidase to MPS I mice. However, improvements were reported to be less profound than when using a gamma retroviral vector with intact long terminal repeats, likely as a consequence of less efficient transduction and expression of the liver [126].

#### Fabry disease

Fabry disease is an X-linked disorder caused by deficiency in α-galactosidase. Undigested glycosphingolipids accumulate in endothelial cells of kidney, heart and liver and symptoms include angiokeratoma, hypohidrosis and episodic pain crises in the extremities with the patient eventually dying of heart or kidney complications. Recombinant enzyme replacement therapy is the main treatment albeit at great financial cost and with immune involvement reducing efficacy . Ogawa and colleagues compared neonatal (2 days) and adult (12 weeks) gene therapy in a mouse model of Fabry disease using AAV1 vector carrying a CAG promoter. Adult gene transfer mainly targeted the liver whereas, following neonatal delivery, expression was concentrated in the heart [127]. In this disease, specific liver gene transfer may not be the preferred target. Furthermore, in this study, AAV1 was quite poor in transducing neonatal tissues therefore the principle advantage of injecting neonates with vector in this study was induction of immune tolerance rather than achieving widespread transduction of affected tissues.

### 3.6 Glycogen Storage Diseases

The glycogen storage diseases (glycogenoses) comprise several genetic deficiencies of enzymes that control glycogen synthesis or catabolism of glycogen. Glycogen is mainly stored in the liver and muscle. Liver-based glycogen storage diseases, which result in hypoglycemia, include glycogen storage diseases Types 0, Ia, Ib, IIIa, IIIb, VI and IX [128]. Clinical manifestations vary depending upon the nature and extent of the deficiency. For example, Type I presents hepatomegaly, failure to thrive, hyperlactatemia, hyperuricemia and hyperlipidemia. Type IV usually presents within 12 months of birth as hepatomegaly and growth retardation and tends to progress to cirrhosis. Management is highly type-dependent but generally involves careful dietary control of glucose levels by glucose infusion, protein feedings, uncooked cornstarch (a means of glucose slow release) [129]. 

Glycogen storage disease type Ia (also known as von Gierke disease) is deficiency of glucose-6-phosphatase. Infants usually present with seizures and hepatomegaly before a year of age. As per other glycogen storage diseases it is managed by glucose infusion and regular (every 3 to 6 hours) dietary uncooked cornstarch. Despite this management, long-term complications include growth failure, renal dysfunction, hypertension and hepatic adenomas that can progress to hepatocellular carcinomas. This disease has received particular attention from gene therapy researchers and studies performed in the early 2000s are reviewed by Koeberl [130]; since only 15% of mice deficient in glucose-6-phosphatase survive until weaning at 21 days the majority of studies were, by necessity, very early interventions. However, it is possible to maintain them until weaning by twice daily intraperitoneal injection of glucose. Administration of early-generation adenoviral vector to 14-day old mice resulted in phenotypic rescue resulting in 90% survival at 84 days of age, however, the authors recognised that expression from this vector was relatively short-lived and that although their results justified further research into gene therapy for this condition, early generation adenoviral vector may not be a suitable candidate for clinical application [131]. It is probably no co-incidence that this sentiment was raised at the same time that a fatality arising from gene therapy of ornithine transcarbamylase deficiency using early generation adenoviral vector was reported [132]. Sun and colleagues co-administered adenovirus and AAV2 vector to 1-2 day old mice by the superior temporal vein and saw correction of the genetic defect and survival beyond one year of age; the mice exhibited normal plasma glucose, cholesterol, triglyceride and uric acid profiles. Interestingly, the authors surmised that the majority of correction could be attributed to the contribution of the AAV vector rather than the adenoviral vector. Nevertheless, the adenoviral vector provided the initial rapid ramping of gene expression that prevented very early neonatal death. Ghosh and colleagues injected single-stranded AAV2/1 into the temporal vein of 1-2 day old knockout mice and some mice received a second infusion, of AAV2/1 or AAV2/8 at 1 week of age. The dual infusion strategy resulted in enzyme expression in liver and kidney for the full 57 weeks of the study [133]. Similarly, Koeberl and colleagues injected single-stranded AAV2/8 vector into 2-week old mice maintained on intraperitoneal glucose and demonstrated a dramatic increase in survival beyond weaning once glucose had been withdrawn [134]. However these studies from 2006 failed to correct, completely, blood glucose levels to those of wild type littermates and very high vector titres were used. Subsequently, Koeberl moved to AAV in a self-complementary format and achieved long-term correction in mice with a 600-fold lower dose of vector. Crucially, it prevented hypoglycemia during fasting in both the mouse and dog models although the dogs had elevated lactate concentrations indicating that correction was not complete. Nevertheless, the study also demonstrated a substantial reduction in mortality in thedog model (after vector injection at 3 days of age) [135]. Weinstein and colleagues also demonstrated correction in dogs which received injection of AAV8 at 1 day of age and a second injection of AAV1 at a later time point. In this study the dogs did achieve normal lactate concentrations. However, results confirmed that a single neonatal injection of AAV8 is not enough to confer correction of glycogen storage disease type Ia [136].

Most of the recent studies have utilised AAV vectors although not to complete exclusion of other vector types. Koeberl and colleagues also injected helper-dependent adenoviral vector into neonatal neonatal mice of a strain deficient in glucose-6-phosphatase. More than half of the treated mice survived beyond 28 weeks in comparison with the untreated group where all mice were dead by 5 weeks of age [137]. In 2010, Feline Immunodeficiency Virus-based lentiviral vector was used to deliver glucose-6-phosphatase to mice deficient in this enzyme. A single neonatal injection corrected neither pathology nor biochemistry however a double neonatal injection protocol, at days 1 and 7, resulted in normalised blood glucose levels substantially extended survival and decreased storage material in the liver. However treated mice still displayed hepatomegaly likely because transduction in the liver was patchy with some areas still showing accumulations of storage material [31].

As a footnote, a distinction should be made between the aforementioned glycogen storage diseases which mainly affect the liver and those which primarily affect the muscle and are reviewed elsewhere [138]. It is also worth pointing out that one notable example of this distinction is glycogen storage disease type II, also known as Pompe disease, which also happens to be a lysosomal storage disease and which presents early in life. Unfortunately, the numerous preclinical studies for gene therapy of this disorder are beyond the remit of this liver-based review. 

### 3.7 Bile Metabolism

Polymorphisms in three genes involved in bilirubin metabolism have been identified as underlying neonatal hyperbilrubinemia. Although generally benign, in severe deficiencies plasma bilirubin concentrations rise to levels that may cause brain damage [139, 140]. One of the more serious genetic defects is of the gene UDP-glucuronosyltransferase 1A1 (UGT1A1). Crigler-Najjar type I disease (CN1) is an autosomal recessive syndrome which results in complete deficiency of the liver enzyme and subsequent unconjugated bilirubin accumulates in the brain resulting in neuropathology and may cause permanent disability. Current treatment is extensive phototherapy which is associated with skin abnormalities and cannot completely prevent brain damage. Therefore, liver transplantation remains the only cure and the case for very early transplantation before the onset of CNS damage is strong [141]. 

Several studies describing fetal or neonatal gene transfer for preclinical models of Crigler-Najjar syndrome have been published. Seppen and colleagues used a lentiviral vector to deliver the gene to a rat model (Gunn rats) of Crigler-Najjar syndrome. Transcutaneous intra-hepatic injection of vector into the fetal liver resulted in long-term partial reduction in serum bilirubin levels. Interestingly, expression was transient in the liver but persisted in other tissues which, the authors pointed out, was clearly sufficient to ensure prolonged gene expression [142]. Interestingly, in a subsequent study, they also observed an immune response against UGT1A1 and suggested that it may be a particularly immunogenic protein [143]. However, the mode of delivery (intraparenchymal injection) may have caused some local tissue damage that precipitated an immune reaction that could be absent with intravascular delivery. In addition, it has been shown that hepatocyte-specific expression of transgene can result in immune tolerance to the protein [144]. Therefore, in achieving prolonged expression in tissues outside the liver but not in hepatocytes, this mechanism would not be triggered. Nguyen and colleagues also used a lentiviral vector for delivery into neonatal Gunn rats. In contrast to Seppen and colleagues, the vector was injected intravenously via the superior temporal vein and hepatocytes were observed to be the main source of expression. In this study, normalisation of bilirubin was achieved for up to at least 95 weeks [145]. The same group used an AAV8 vector to treat neonatal Gunn rats. In this case, serum bilirubin concentrations were only reduced transiently suggesting that this would not be a viable therapeutic approach. 

### 3.8 Immune Diseases

Two interesting studies have used gene delivery to the liver as a means of addressing immune system defects. In the first, Carbonaro and colleagues used an HIV-1 based lentiviral vector for expression of adenosine deaminase (ADA) in ADA-deficient mice following neonatal intravenous injection. ADA enzyme activity was significantly lower in thymus and spleen than found in wild-type mice but was elevated between 2-10 fold in livers of treated mice compared with wild-types and 10-100 fold greater than found in the knockouts; proviral copy number was mainly detected in the liver. Immune function was greatly improved by this strategy and the authors surmised that the lymphoid system may have been rescued *in trans* by metabolic effects of ectopic ADA expression. They suggested that the liver was acting as a ‘sink’ for degradation of deoxyadenosine and adenosine nucleosides or that circulating ADA, likely secreted by the liver, was being distributed systemically [146]. In the second study, Spitzer and colleagues used gamma retroviral vectors in the treatment of a mouse model deficient in C receptor 1-related gene/protein y (Crry). They designed and delivered a retroviral vector expressing Crry with single chain antibody fragment which ensured that secreted Crry could attach to, and correct the deficiency on the surface of red blood cells. They demonstrated that this strategy was able to correct the complement activation defect in these mice for up to at least a year [147], illustrating the concept of using the liver as a protein factory.

## 4 CHALLENGES AND PERSPECTIVES

### 4.1 Genotoxicity and Oncogenicity Risks of Early Gene Transfer

There is some evidence that gene transfer in the fetal or neonatal period engenders a higher risk of genotoxicity or even malignant transformation. We observed a high incidence of hepatocellular carcinomas and the occurrence of metastatic events following fetal and neonatal intravenous, intramuscular and intraspinal injection of EIAV-based lentivirus vector for delivery of β-galactosidase and human factor IX [23]. We have observed a high incidence of tumor formation following fetal intravenous injection with FIV-based lentivirus in some (unpublished observations) but not all studies [31]. However, to date, following several studies where many mice were injected *in utero* and neonatally by these routes with HIV-based lentivirus we have observed no increase, over background, in the incidence of hepatocellular carcinoma [34, 67, 148]. Tittiger and colleagues also observed no increase in oncogenesis following early gene transfer of gamma retrovirus vectors [149]

Studies by Donsante and colleagues have also reported a high incidence of hepatocellular carcinoma in AAV-treated mice with lysosomal storage disease [150] and have implicated insertional mutagenesis as the causative mechanism [151]. However several subsequent studies have failed to observe tumor formation following neonatal delivery of AAV vectors at very high titres into neonatal or fetal mice [152-154].

### 4.2 Advantages and Disadvantages of Fetal and Neonatal Gene Transfer to the Liver

The advantages of early liver gene transfer (or, to any other organ system) have been restated in numerous reviews and published studies over the past decade or more on fetal [1, 2, 73] and neonatal [71, 155] gene transfer. Firstly, since the fetal or neonatal organism is small and contains much fewer cells it is possible to achieve a much higher vector:cell ratio with early intervention. Secondly, stem cell and/or progenitor populations are likely much more accessible early in life due to their abundance and their physical placement in the developing tissues/organs. Thirdly, early gene transfer and, thus, expression may predispose the immune system towards immune tolerance of the expressed xenoprotein; as Billingham, Brent and Medawar demonstrated in the early 1950s; the developing immune system undergoes a period where it becomes tolerant to self antigens [156]. One of the major hurdles facing adult gene therapy is the development of an immune response that eliminates transgene expression. However, there are numerous aspects to this response and the advantage which early gene transfer provides. Early transgene expression may persuade the immune system that the expressed protein is “self”. A *de novo* immune response to capsid proteins, such as seen in the recent adult trial for hemophilia B [54] may be avoided since the neonatal adaptive immune response is thought to be relatively poor [157]. The individual may have been exposed to antigenic epitopes of the wild-type virus from which the vector is derived. This would be avoided by early gene transfer to a recipient whose immune system was almost completely naive to infection. The fourth and, likely, most important advantage of early intervention is that it could prevent or ameliorate the onset of early and irreversible pathological changes. 

The area of fetal and neonatal gene transfer to the liver (and other organ systems) faces several conundrums. 

Firstly, is the question of whether gene transfer before birth would ever be carried out in preference to postnatal intervention. This would depend upon several things. For diseases where pathological changes occur later in infant life, for example hemophilia, neonatal gene transfer may be adequate. However other diseases, such as severe Protein C deficiency [158] and OTC deficiency [88], may manifest severe pathology even before birth. In these cases it would be essential to intervene before pregnancy had come to term: One alternative to *in utero* intervention would be elective early delivery and injection into the neonate. An interesting article by Pollock-BarZiv describes 26 fetuses that were diagnosed with profound heart defects and were listed as amenable for heart transplantation. Two of these fetuses were surgically delivered at 36 weeks gestation when a donor organ became available and were transplanted within the first six hours of life [159]. A second consideration is the fundamental changes that occur in the liver from fetal to neonatal life. In humans hematopoiesis in the liver peaks around 22 weeks gestation which then declines thereafter as the liver gradually assumes its phenotype of postnatal life; in mice it peaks at 14.5 days gestation [160]. Since the fetus gains nutrients from the placenta, portal circulation from the intestines to the fetal liver is less important than in adult life and the *ductus venosus* acts as a shunt permitting a substantial fraction (20-50%) of umbilical blood flow to bypass the liver and to be delivered directly to the heart. At birth profound circulatory changes induce closure of this vessel [161]. Whether either the hematopoietic or circulatory differences between the fetal and neonatal liver are relevant to efficacy of gene targeting is debatable. Unless injected as an asanguinous bolus, vector particles might be expected to transduce cells with cognate receptors over several passes through the entire circulation. Similarly there is no evidence to suggest that hematopoietic cells populating the fetal liver are any more amenable to transduction than when they assume residence in the bone marrow where they remain through postnatal life. 

A second question relates to the issue of whether serious genetic diseases could ever be diagnosed early enough to provide prophylactic gene therapy. For families with affected siblings genetic screening would be feasible. However, in many cases the underlying cause is a *de novo* mutation. An interesting perspective is provided in a study by Hayes and colleagues of adults and of parents of individuals with mucopolysaccharidoses. They conducted a survey to assess hypothetical clinical scenarios about newborn screening for mucopolysaccharidosis which is now technically possible. Ninety-seven percent supported the use of newborn screening in situations where early treatment for the disease was available but 87% supported newborn screening for severe mucopolysaccharidosis where no treatment was available. The most common reason cited for this was that newborn screening could avoid the stress of delayed diagnosis [162].

**Fig. (2):**
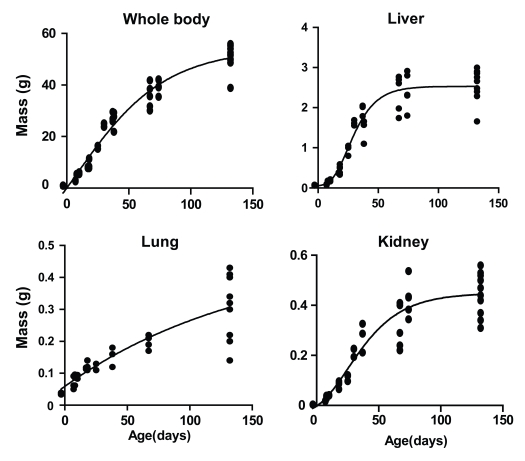
Whole body weight and mass of liver, lung and kidney in MF1 outbred mice in the first 150 days of life.

A third question is, “Which is the best vector?”. It has become apparent that each vector has core strengths and applications for clinical gene therapy of adult patients. For example, AAV has been the most effective in topical delivery to the retina for inherited blindness [163-165] and also for liver-targeted treatment of hemophilia [53, 56]. Gamma retroviral and lentiviral vectors have performed well for *ex vivo* gene therapy of hematopoietic stem cells for treatment of X-SCID [166], ADA-SCID [167] and X-linked Adrenoleukodystrophy [168]. Adenovirus has been applied extensively for anti-cancer therapy. A summary of selected current gene therapy clinical trials, categorised by vector is provided by Sheridan [169]. For neonatal gene therapy of the liver several factors need to be considered. The opinion of these authors is that adenovirus vector in either a first or second generation format or even a helper-dependent format is unlikely to be first choice for treatment of an inherited liver disease. Concerns over immune responses towards capsid proteins following systemic adenovirus delivery remain even though these barriers have been shown to be surmountable in a preclinical setting [170]. Therefore the choice is between retroviral vectors (including lentivirus vectors) and AAV. The increase in mass of the human liver is relatively linear from 12-34 weeks gestation but around birth demonstrates an upswing in growth [171] likely representing the acquisition of metabolic responsibility concomitant with loss of maternal metabolic support at birth. Therefore retrovirus, which integrates into the host genome, should maintain parity of expression with host proteins as the liver grows. In contrast AAV vector, which is generally considered to be episomally maintained, should be diluted out during rapid cell proliferation in the maturing liver. As shown in Fig. **2**, the mouse liver has reached nearly maximum mass by 70 days of age whereas other organs such as lung and kidney as well as whole body weight are still increasing. Similarly in the early neonatal period in humans there is a relatively large increase in liver mass compared with other organs [171]. These phenomena are highlighted eruditely in the comparison of vectors reviewed by Ponder and Haskins [108]. They are also demonstrated clearly in fetal and neonatal studies using AAV and lentivirus vectors: When lentivirus was used for fetal and neonatal treatment of hemophilias A [67] and B [66], respectively, plasma coagulation factor concentrations remained relatively steady despite a huge increase in liver mass in the first hundred days of life. In contrast, AAV vectors applied to mice [152], sheep [74] and non-human primates [75] resulted in a dramatic loss of expression. In mice this loss was near total whereas in non-human primates the levels fell to levels that remained at physiological concentrations in one individual. For hemophilia, a residual concentration of even >1% would convert a severe hemophilia into one classified as moderate and >5% would be classified as mild [172] therefore neonatal AAV might suffice. In contrast, gene therapy for mucopolysaccharidosis type I may require several-hundred fold increase (over normal) in enzyme activity, secreted by the liver, to correct the systemic defect [108] and this may only be achievable using gamma retroviral vectors. Ultimately, success of AAV in adult clinical trial for systemic gene therapy is likely to be the strongest vindication of candidacy as a viable vector for fetal or neonatal gene transfer. Yet the efficiency of AAV to cross vascular and cellular barriers may yet, for fetal gene transfer, have unwanted side effects. Mattar and colleagues have demonstrated, following AAV8 and AAV5 administration to fetal macaque, transplacental transfer from fetus to mother [75]. 

## 5 ENDNOTE

The numerous and recent references cited in this review illustrate and emphasise that the technology for treating serious genetic liver diseases is reaching maturity and fetal or neonatal application of this technology is both feasible and necessary. The Titan Prometheus, of Greek mythology, was known as champion of mankind who gave the Zeus’ fire to the mortals. Zeus punished him by tying him to a rock; a huge eagle tore out his liver every day but every night it grew back, to be eaten again the following day [173]. Recent advances in technology give us hope that we may soon have genetic therapeutic interventions for the most devastating of diseases which beset the liver. Combating liver-munching eagles with gene therapy may take a little more time.

As a final note it is with regret that through space concerns we have omitted many other important studies and we would like to apologise to the authors of these studies.
